# Moving Time: The Influence of Action on Duration Perception

**DOI:** 10.1037/a0037650

**Published:** 2014-08-04

**Authors:** Clare Press, Eva Berlot, Geoffrey Bird, Richard Ivry, Richard Cook

**Affiliations:** 1 Department of Psychological Sciences, Birkbeck, University of London; 2 Division of Psychology and Language Sciences, University College London; 3 MRC Social, Genetic and Developmental Psychiatry Centre, Institute of Psychiatry, King’s College London; 4 Department of Psychology, University of California, Berkeley; 5 Department of Psychology, City University London

**Keywords:** motor processes, perceptual motor coordination, time perception, social perception

## Abstract

Perceiving the sensory consequences of action accurately is essential for appropriate interaction with our physical and social environments. Prediction mechanisms are considered necessary for fine-tuned sensory control of action, yet paradoxically may distort perception. Here, we examine this paradox by addressing how movement influences the perceived duration of sensory outcomes congruent with action. Experiment 1 required participants to make judgments about the duration of vibrations applied to a moving or stationary finger. In Experiments 2 and 3, participants judged observed finger movements that were congruent or incongruent with their own actions. In all experiments, target events were perceived to be longer when congruent with movement. Interestingly, this temporal dilation did not differ as a function of stimulus perspective (1st or 3rd person) or spatial location. We propose that this bias may reflect the operation of an adaptive mechanism for sensorimotor selection and control that preactivates anticipated outcomes of action. The bias itself may have surprising implications for both action control and perception of others: we may be in contact with grasped objects for less time than we realize, and others’ reactions to us may be briefer than we believe.

To interact appropriately with physical and social environments, actors must predict and evaluate the sensory consequences of their actions. We select actions on the basis of their predicted outcomes (Greenwald, 1970; Hommel, Müsseler, Aschersleben, & Prinz, 2001), and when the experienced sensory information deviates from the prediction, corrective adjustments can be made to ensure successful execution. For example, when picking up a cup of tea, the motor commands generated result in both visual (e.g., sight of grasping and lifting) and tactile (e.g., pressure on the fingertips) sensory consequences. If the actual feedback differs from the anticipated sensory outcomes, rapid corrective actions can be executed to avoid spillage. Similarly, when interacting with others, rapid response prediction and error correction may facilitate smooth social interactions (Wolpert, Doya, & Kawato, 2003).

Successful interaction with the environment requires perception of not only the nature of our action outcomes (e.g., somatosensation on the fingertips during grasping) but also, crucially, the onset and duration of those outcomes. We are sensitive both to the “what” and “when” of sensory predictions (Blakemore, Frith, & Wolpert, 1999; Blakemore, Wolpert, & Frith, 1998; Christensen, Ilg, & Giese, 2011; Fagioli, Hommel, & Schubotz, 2007; Hommel, 2010; Lee, Young, Reddish, Lough, & Clayton, 1983; Schubotz, 2007). For example, lifting the teacup from a saucer requires an anticipatory response to maintain postural stability (Diedrichsen, Verstynen, Hon, Lehman, & Ivry, 2003; Dufossé, Hugon, & Massion, 1985). Similarly, anticipating the duration of the lift phase is essential for coordinating hand and mouth gestures, and when shaking someone’s hand, contact must be made for an appropriate length of time—neither too long nor too short—to convey the intended social message.

While the preceding examples underscore the importance of temporal information in the generation and perception of sensory expectancies, duration perception is frequently distorted. For example, perceived motion of upright point light walkers is temporally dilated relative to inverted walkers (Wang & Jiang, 2012; see also Gavazzi, Bisio, & Pozzo, 2013). In the present experiments, we examine how movement influences the perceived duration of sensory outcomes of action. Sensory prediction mechanisms essential for action selection and fine-tuned control may, paradoxically, distort the perceived duration of outcomes, with consequences for action control and perception in a variety of contexts. In Experiment 1, participants were required to perform a lifting movement with either their index finger or their middle finger. A short target vibratory tactile stimulus was presented to the moving or stationary finger, followed by a second reference vibration. Participants judged which was of longer duration, allowing us to determine how action influences duration perception. In Experiments 2–4, we investigated whether action influences the perceived duration of visual events in a comparable manner.

## Experiment 1

Sixteen right-handed, healthy adults (12 men) with a mean age of 29.3 years (standard error of the mean [*SEM*] = 2.5) participated in the experiment. Three were replacements for participants who could not perform the perceptual discrimination (psychometric functions could not be modeled or their point of subjective equivalence [PSE] fell outside the range of presented stimuli). All experiments were undertaken in accordance with the 1964 Declaration of Helsinki.

**Figure 1 fig1:**
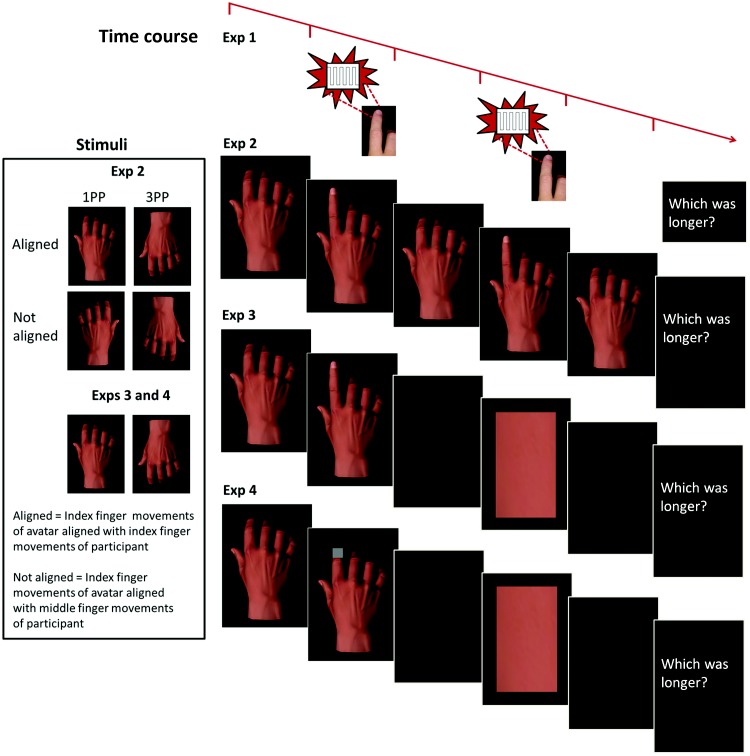
The visual stimuli (created using Smith Micro Software’s Poser 7.0) and time course for the action-related events in each of the four experiments. Time course stimuli depict the avatar hand in first-person perspective. See the online article for the color version of this figure. Exp = experiment; 1PP = first-person perspective; 3PP = third-person perspective.

The experiment was conducted in MATLAB using the Cogent toolbox.^1^ Two 5V solenoids, each driving a metal rod with a blunt conical tip (diameter ≈ 1.5 mm, skin indentation ≈ 1 mm), were attached to the distal phalange (ventral surface) of the index and middle fingers on the participant’s right hand. Participants held down two keys on the keyboard until an imperative cue instructed them to lift either their index (*I*) or their middle (*M*) finger. They were instructed to make large, rapid, single-movement lifts. Their response hand was visually occluded. Approximately 10 ms after the cued finger was lifted, a target vibration lasting for one of seven durations (104–296 ms, 32-ms steps) was applied to the moving (congruent) or stationary (incongruent) finger (see Figure 1). After an interstimulus interval (300–500 ms), a 200-ms reference vibration was applied to the same finger. Both vibratory stimuli were presented at 62.5 Hz.[Fig-anchor fig1]

Participants judged whether the target or reference vibration was longer, responding with a button press made with their left hand. After this response, they returned the lifted finger to the start position. The next trial started after 2,000 ms. There were 280 trials: 140 in which stimuli were applied to the congruent finger and 140 where they were applied to the incongruent finger. Trial type was randomized, and participants completed eight practice trials.

**Figure 2 fig2:**
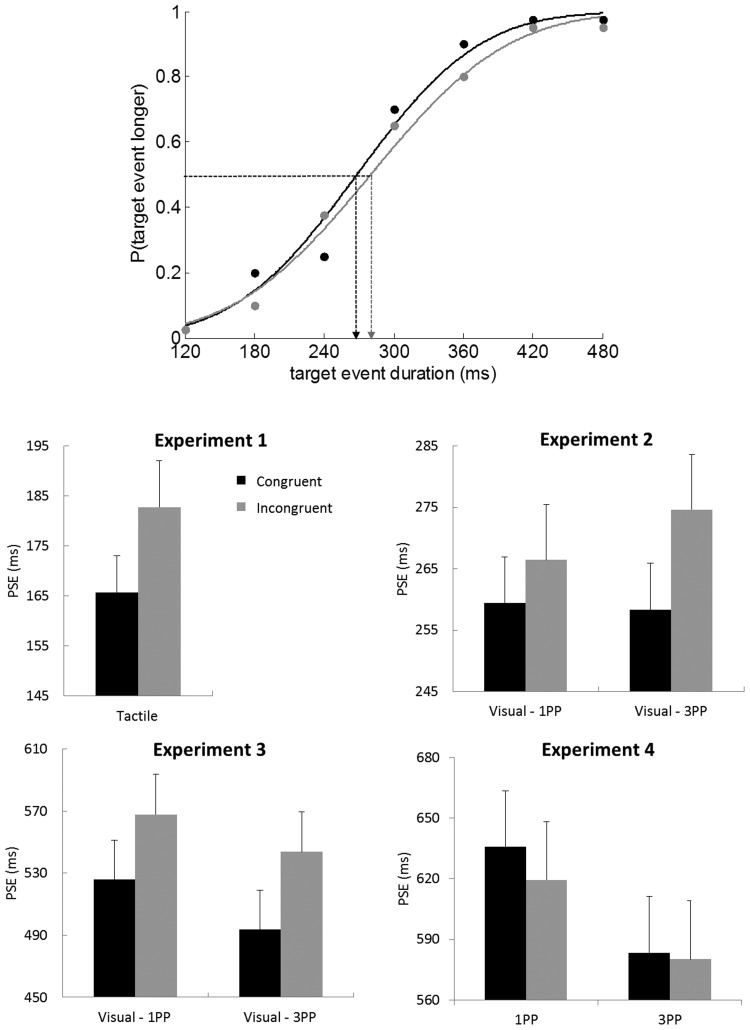
Top panel: Demonstration of how the point of subjective equivalence (PSE) was calculated with psychometric functions for an example participant, with stimuli congruent and incongruent with moving fingers. The PSE describes the point where participants judge the target and reference events to have equal duration. Judgment precision was inferred from the standard deviation of the Gaussian distribution that best fits the data; it pertains to the inverse of the slope, and lower thresholds reflect more consistent categorizations, thereby indicating better performance. Other panels: Mean PSEs for stimuli congruent and incongruent with moving fingers, for all experiments and perspectives. 1PP = first-person perspective; 3PP = third-person perspective. Error bars represent the standard error of the mean.

To estimate psychometric functions, the responses for each individual were modeled by fitting cumulative Gaussians, and associated pDev statistics were calculated to establish the goodness of fit of each function (Palamedes toolbox; Kingdom & Prins, 2010). This procedure was performed separately for congruent and incongruent response data. In each condition, bias was inferred from the PSE and precision from the difference threshold (see Figure 2).[Fig-anchor fig2]

The participants were more precise in their judgments when the vibratory stimuli were applied to the congruent relative to incongruent finger, *t*(15) = 2.3, *p* < .05, η^2^ = .26; see Table 1. There was also an effect on PSE: Target events were judged to be longer when the stimulus was applied to the congruent relative to the incongruent finger, *t*(15) = 2.6, *p* < .02, η^2^ = .32 (see Figure 2 and the supplemental materials). In sum, tactile events presented to a moving effector are perceived to be longer and are judged more consistently than when that effector is stationary.[Table-anchor tbl1]

## Experiment 2

If prediction mechanisms operate in social contexts, we may predict and evaluate sensation related to not only our own actions but also actions produced by interactants (Wolpert et al., 2003). As such, we should observe comparable action-related predictive modulation with visual action stimulus events. Additionally, such mechanisms should operate across perspectives given the range of viewpoints from which others’ actions are observed. Therefore in Experiment 2 we examined duration perception of congruent and incongruent visual events during action, across stimuli presented from first- and third-person perspectives.^2^

Sixteen right-handed, healthy adults (12 men) with a mean age of 25.9 years (*SEM* = 1.9) participated in the experiment. Five were replacements for participants who could not perform the discrimination. Unless otherwise stated, procedural information already outlined in Experiment 1 is identical in this, and all subsequent, experiments. Participants compared the duration of two finger movements simulated visually by gestures of an avatar hand. At the start of the trial, the avatar hand was presented in a neutral position on the monitor (see Figure 1; screen refresh rate = 85 Hz). An imperative cue (*1* or *2*) was presented between the index and middle fingers. When participants lifted the cued finger, the neutral hand image was immediately replaced (within the constraints of the refresh rate) by one depicting the avatar hand performing either an index finger or a middle finger lift for 120–480 ms (seven levels; 60-ms steps). This resulted in apparent motion of the avatar’s finger approximately synchronized with the participant’s action. At the offset of the target event, the avatar hand resumed the neutral position for an interstimulus interval of 300–500 ms, followed by a second image of the same lifted finger for a reference duration of 300 ms and then the neutral image again for 300 ms. Participants judged which lift lasted longer. The range of durations was chosen to match discrimination performance in Experiment 1.

There were four block types. In spatially aligned first-person perspective (1PP) blocks, participants viewed a right avatar hand with fingers aligned in the horizontal plane with their own right hand (see Figure 1). In spatially aligned third-person perspective (3PP) blocks, the avatar hand was rotated about the horizontal axis (therefore presenting a left hand). The remaining blocks consisted of these stimuli flipped on a vertical axis, such that corresponding finger movements did not match in spatial location (necessitating a left hand for 1PP and a right hand for 3PP). These blocks thereby controlled for the spatial location of finger movement (Press, Gherri, Heyes, & Eimer, 2010). The four blocks each comprised 140 trials and were completed in a counterbalanced order.

The precision and PSE distributions were analyzed using separate three-way ANOVAs, with factors of movement congruency (avatar and participant moved the congruent or incongruent finger), location (avatar and participant finger movements were made in aligned or misaligned horizontal locations), and perspective (1PP or 3PP). No precision effects were observed (all *F*s < 2.1, all *p*s > 0.17; see Table 1). However, as in Experiment 1, target events were perceived to be longer when the avatar and participant moved the same finger, *F*(1, 15) = 5.3, *p* < .04, η^2^ = .26). There were no other main effects or interactions (all *F*s < 2.5, all *p*s > 0.14). These results indicate a bias to judge target events to be longer when observed actions are congruent with self-generated actions, regardless of whether stimuli are observed from first- or third-person perspectives. Notably, these effects reflect congruency between effectors (same finger) rather than spatial location.

## Experiment 3

Experiment 2 suggests that action performance influences the perceived duration of effector-congruent visual events. However, it is possible that despite informing participants that the reference event was always presented for the same length of time, participants’ actions might have influenced the perceived duration of the reference rather than the target event. To control for this possibility, we modified the reference event in Experiment 3. Rather than define the reference duration by a second avatar movement, this interval was defined by the stimulus duration of a rectangle, a neutral stimulus selected because it exhibited no congruency relationship with the fingers.

Sixteen right-handed, healthy adults (11 men) with a mean age of 28.3 years (*SEM* = 2.2) participated in the experiment. Three were replacements for participants who could not perform the discrimination. The imperative cue (*I* or *M*) was presented between the index and middle fingers of the observed hand. When participants lifted the cued finger, the neutral hand image was immediately replaced by an image of a hand with a lifted index or middle finger for 150–900 ms (seven levels; 125-ms steps). After an interstimulus interval of 300–500 ms, a rectangle was presented for a reference interval of 525 ms. The color, luminance, and aspect ratio of the rectangle were identical to that of the avatar hand. The test stimulus range was selected on the basis of pilot testing to yield comparable performance to that observed in Experiments 1 and 2. Participants again judged which of the two intervals was longer. Given that spatial location had no impact on the effect in Experiment 2, only aligned blocks were included. Participants completed 1PP and 3PP blocks, each comprising 140 trials, in a counterbalanced order.

The precision analysis yielded no main effects or interactions (all *F*s < 1.4, all *p*s > 0.25; see Table 1). However, the PSE phenomenon observed in Experiments 1 and 2 was replicated: Target events were perceived to be longer when the observed event was congruent with the participant’s action, *F*(1, 15) = 6.5, *p* < .03, η^2^ = .30 (see Figure 2). As in Experiment 2, this effect did not interact with perspective, *F*(1, 15) = 0.05, *p* = .8, η^2^ = .02. These findings demonstrate that action biases perception of the temporally contiguous target event, rather than reference events presented after a delay.

## Experiment 4

It is possible that the temporal dilation effects in Experiments 2 and 3 result from attentional orienting toward the location of the congruent effector. Increased attention may influence the perceived duration of events at this location irrespective of action–stimulus congruency. We conducted a final experiment to test this possibility, which was identical to Experiment 3 except that target durations were defined by the presentation of a rectangle over the fingertip of the index or middle finger rather than by a finger movement (see Figure 1). If attentional orienting generates temporal dilation effects irrespective of the nature of the target event, similar influences of congruency will be observed.

Sixteen right-handed, healthy adults (seven men) with a mean age of 27.7 years (*SEM* = 1.7) participated in the experiment. One was a replacement for a participant who could not perform the discrimination. The precision analysis yielded no main effects or interactions (all *F*s < 0.7, all *p*s > 0.41; see Table 1). Unlike Experiments 1–3, there was also no PSE effect, *F*(1, 15) = 0.7, *p* = .42 (see Figure 2). An ANOVA conducted on the PSE data from both Experiments 3 and 4 with experiment as a between-subjects factor revealed a Congruency × Experiment interaction, *F*(1, 30) = 6.8, *p* < .02, η^2^ = 0.2. These results argue against this attentional orienting account of the congruency-induced temporal dilation.

## Discussion

The present findings demonstrate a bias to judge sensory events to be longer when they are congruent with a concurrently performed action. This effect was found when participants judged the duration of tactile vibrations applied to a moving finger, as well as when assessing the duration of observed finger movements.^3^ These results indicate that subjective action time can be subject to temporal dilation: Events effector congruent with performed actions are perceived to be longer than events incongruent with those actions.

These effects may be a consequence of preactivated action expectancies during selection and preparation (Greenwald, 1970; Hommel et al., 2001), whereby congruent sensory events are perceived to begin before action onset. Imperfect distinctions between anticipated and actual sensory consequences would cause congruent sensation to be perceived as longer. In contrast, when action consequences are unexpected, preactivated outcomes differ from the actual sensory consequences and can thus be discriminated. The hypothesis that duration biases result from imperfect distinctions between predicted and stimulus-driven percepts is consistent with the finding that imagined and perceived visual events activate common occipital representations (Albers, Kok, Toni, Dijkerman, & de Lange, 2013; Kosslyn et al., 1993; see also Bueti & Macaluso, 2010) and that action preparation activates representations of the anticipated effects (Kühn, Keizer, Rombouts, & Hommel, 2011; Müsseler & Hommel, 1997). Furthermore, the idea that the perceived onset of anticipated events is shifted in time is consistent with a number of temporal distortions in the action control literature. For example, it has long been recognized that when tapping to a metronome, movements show a phase lead to the pacing signals (Bartlett & Bartlett, 1959; Dunlap, 1910). Moreover, effects resulting from action but at delay are perceived to occur earlier than in reality (Haggard, 2005).

Temporal biases resulting from the prediction of congruent action consequences might be expected to detract from effective action control. However, illusory biases often result from the operation of adaptive mechanisms. For example, visual aftereffects, defined by significant sensory distortion, are believed to be the products of ongoing perceptual recalibration to ambient sensory inputs (Clifford & Rhodes, 2005; Thompson & Burr, 2009; see also Yarrow, Haggard, Heal, Brown, & Rothwell, 2001). Similarly, stimulus-general temporal dilation during action planning may maximize information acquisition prior to movement (Hagura, Kanai, Orgs, & Haggard, 2012). Following this line of reasoning, we suggest that the dilation of subjective action time observed for anticipated sensory outcomes may be indicative of an adaptive mechanism optimized for online action control. Anticipation of the sensory consequences of action is essential for action selection and subsequent error correction. Imperfect distinction between anticipated and actual sensory outcomes may reflect exploitation of mechanisms adapted for perception during action planning. Although these mechanisms broadly benefit actors, there may be surprising consequences for tightly time-locked action control and social perception. For example, we may be in contact with grasped objects for less time than we realize and handshakes may be briefer than we believe.

Equivalent effects when observing sensory events from first- and third-person perspectives suggests that common mechanisms may anticipate the consequences of one’s own actions as well as the imitative reactions of others. Wolpert et al. (2003) proposed that sensory prediction mechanisms for action control may also operate when interacting with others, but this possibility has received little empirical investigation. The present study provides support for this hypothesis, suggesting that we overestimate the duration of not only our own actions but also others’ imitative reactions. Future investigations must establish whether these effects are found when other individuals react in a nonimitative but predictable manner, for example, when dominant body postures result in complementary submissive postures of an interactant (Tiedens & Fragale, 2003).

Neuropsychological and neuroimaging studies have implicated motor structures in duration perception, even when action is not required. For example, the cerebellum and basal ganglia are thought to play key roles in a range of temporal judgments (Harrington, Haaland, & Hermanowicz, 1998; Ivry & Keele, 1989; Ivry, Spencer, Zelaznik, & Diedrichsen, 2002; Koch et al., 2007). Additionally, greater activation has been observed in cortical motor areas, including the supplementary motor area and dorsal premotor cortex, when judging the duration of visual events (Coull, Nazarian, & Vidal, 2008; Ferrandez et al., 2003) than when making intensity or color judgments about the same stimuli. These duration judgments may recruit the motor system to exploit mechanisms adapted, either phylogenetically or ontogenetically (Heyes, 2003), for action control.

## Supplementary Material

10.1037/a0037650.supp

## Figures and Tables

**Table 1 tbl1:** Mean Precision Estimates for Stimuli Congruent and Incongruent With Moving Fingers

Experiment and stimulus type	Congruent		Incongruent	
	*M*	*SEM*	*M*	*SEM*
Experiment 1: Tactile	107.5	52.3	129.4	68.5
Experiment 2: Visual—1PP	100.6	13.5	100.2	12.1
Experiment 2: Visual—3PP	100.5	14.5	111.3	14.8
Experiment 3: Visual—1PP	379.9	67.1	346.8	48.2
Experiment 3: Visual—3PP	330.3	40.6	287.3	35.2
Experiment 4: 1PP	294.2	21.9	283.1	28.6
Experiment 4: 3PP	318.1	36.5	319.5	37.7
*Note.* 1PP = first-person perspective; 3PP = third-person perspective.				

## Footnotes

1 John Romaya, Wellcome Trust Centre for Neuroimaging and Institute of Cognitive Neuroscience development team, University College London.

2 Examining congruency-induced temporal dilation in the visual modality also permits better isolation of perceptual effects from the direct effects of action performance. Visually defined congruency is eliminated when the hands are occluded. It is not possible to eliminate tactile-defined congruency without some form of sensory deafferentation.

3 This similarity was observed across experiments despite changes in the range of durations presented. It is worth noting that piloting indicated these shifts in duration to be necessary for two reasons. First, the apparent motion in Experiments 2 and 3 did not appear natural with short durations. Second, the duration judgments became more difficult across experiments, moving from punctate touch to apparent motion in vision in Experiment 2 and changing the nature of the reference relative to the target in Experiment 3. Given these changes to the durations presented in Experiments 1–3, it is difficult to draw conclusions concerning the presence of a precision effect in Experiment 1 and its absence in Experiments 2 and 3.
