# Supplementary material for: Moving Time: The Influence of Action on Duration Perception
Source: J Exp Psychol Gen. 2014 Aug 4;143(5):1787–93. doi: 10.1037/a0037650 (PMC4170821; doi:10.1037/a0037650)
Supplement: Supplementary file 1 [file XGE-XGE3-Press20131717-R-F3.pdf]

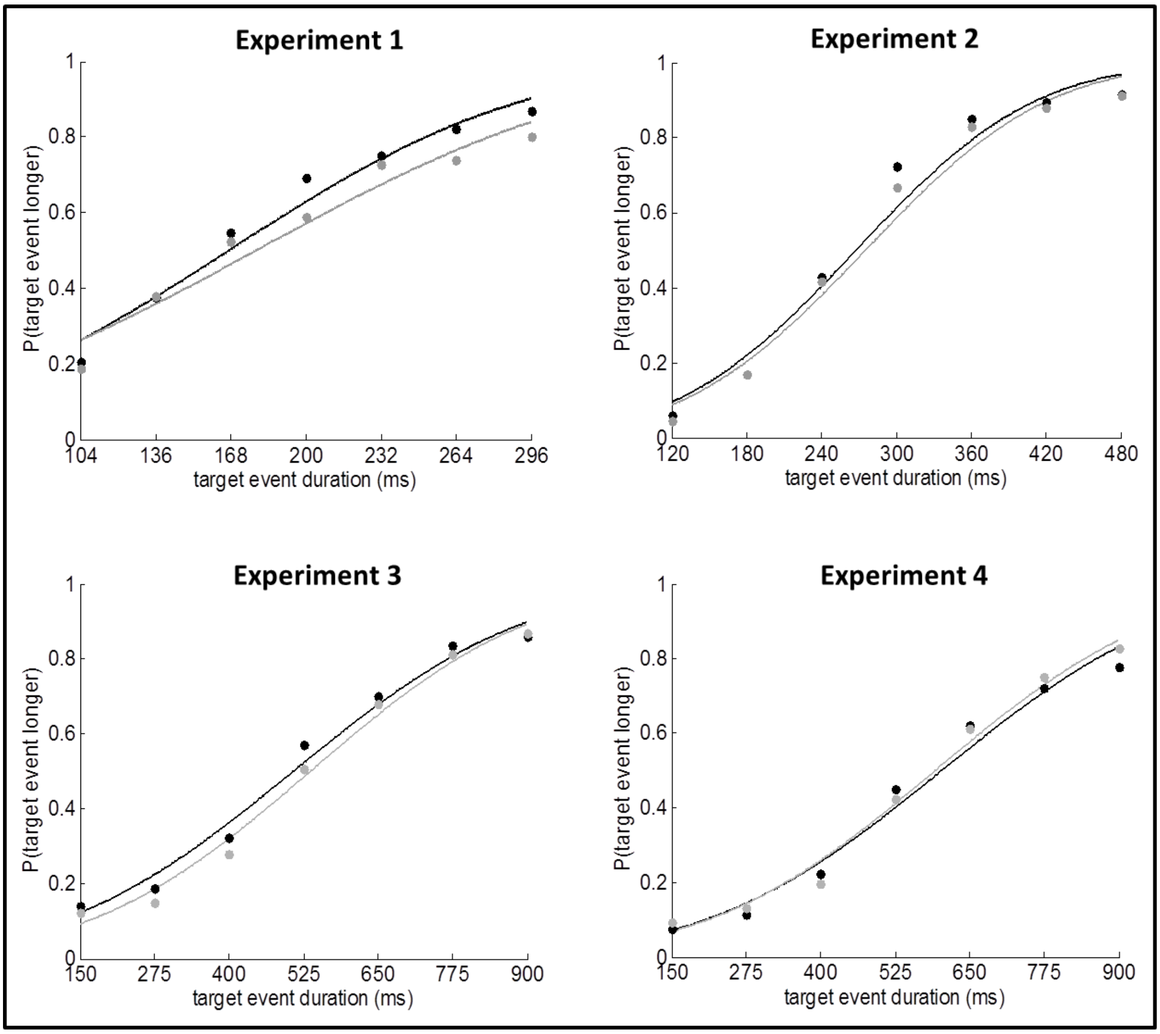

Supplementary Figure 1. Mean responses at each of the seven levels of target stimulus duration, for stimuli congruent and incongruent with moving fingers in all experiments. Congruent datapoints are represented in black and grey points show the incongruent data. Psychometric functions are modeled for representational purposes.
